# Emergency Department Navigator Interventions and Outcome Measures: A Scoping Review

**DOI:** 10.1111/opn.70026

**Published:** 2025-03-31

**Authors:** Kathleen Parry, Christopher Picard, Rashmi Devkota, Kaitlyn Tate

**Affiliations:** ^1^ Alberta Health Services Edmonton Alberta Canada; ^2^ Nursing, College of Health Sciences University of Alberta Edmonton Canada

**Keywords:** emergency department, interventions, older people, primary care, scoping review, transition

## Abstract

**Introduction:**

Emergency department (ED) patient navigators are increasingly used, but a lack of understanding of how ED navigator interventions are designed, described, and evaluated creates gaps in our ability to understand, monitor and improve care. The purpose of this scoping review is to identify how the literature describes and evaluates ED patient navigator interventions for older people transitioning to a primary care setting.

**Methods:**

A scoping review was conducted following the Johanna Briggs Institute updated methodological guidance for the conduct of scoping reviews. We searched three databases: MEDLINE, EMBASE and CINAHL. We included English language articles without any restrictions on study designs that two reviewers screened. All articles focused on distinct ED navigator roles to facilitate transitions for older people from the ED to primary care were included. Data extraction was completed by the primary reviewer and validated by two secondary reviewers. We report study characteristics in a table. Descriptive content analysis was used to analyse the main findings.

**Results:**

A total of 10 studies were included out of 2102 articles identified. All studies used quantitative designs except one, which used a qualitative research design. Four studies were conducted in the United States, two in Australia and the UK and one in Canada and Belgium. Twenty unique outcome measures were identified, with hospital admission rate, frequency of ED presentations and ED/hospital length of stay being the most common. We identified six intervention components: assessment, consultation, liaison, development of care plan, referral and follow‐up. Interventions using 4 or more components more commonly reported positive outcomes. Outcome measures used to evaluate interventions were often not tracked across care settings, potentially obscuring the impact of ED navigator interventions across the care continuum.

**Conclusion:**

Future research should examine which patients benefit from ED navigation and which outcome measures might help contextualise intervention effectiveness across care settings.

**Summary**

- What does this research add to existing knowledge in gerontology?
  ○ This review addresses the existing knowledge gap on ED navigator interventions for the care transition of older people and the outcome measures used to evaluate its effectiveness.
  ○ There is a lack of consistency among studies in the intervention design and components included to facilitate care transitions for older people from the ED to the community.
- What are the implications of this new knowledge for nursing care for and with older adults?
  ○ Nurses can evaluate the effectiveness of ED navigator intervention using multiple measures to uncover contextual issues.
  ○ We emphasise the importance of measuring the impact of the ED navigator role across care settings and using a team‐based approach.
- How could the findings be used to influence practice, education, research, and policy?
  ○ More research to identify outcome measures that can contextualise intervention effectiveness across care settings is needed to guide the improvement of older people's care transitions.
  ○ The findings from this review highlight the need to create and implement ED navigator roles that meet the transition care needs of older people.

## 1 Introduction

Older people (≥ 65 years) represent almost 25% of the 15.4 million ED visits in 2022/2023 across Canada (Canadian Drug and Health Technology Agency 2023). Most often, older people who visit the ED have complex, specialised medical needs, a higher comorbidity index and significant polypharmacy (Dufour et al. 2020). An older person who enters the ED can expect to experience multiple care transitions and care providers, which may add significant challenges in transitioning to or from the ED (Bambach and Southerland 2021). Various system, provider and patient‐level factors can complicate the transitions of care (Bambach and Southerland 2021), resulting in adverse consequences such as medication errors, interactions or side effects, ED representation or hospital admission. System factors may include but are not limited to care fragmentation, ED overcrowding, inadequate staffing and barriers in accessing outpatient resources (Scott et al. 2017). Provider‐related factors include factors such as gaps in communication between ED and other healthcare professionals leading to incomplete or conflicting information to patients, minimal planning time for ED discharge and low engagement with community partners (Apker et al. 2007; Lennox et al. 2019). Patient‐level factors such as lack of social support, complex comorbidities, communication difficulties and low health literacy leading to gaps in understanding of their health/follow‐up instructions can all significantly impact an older person's ability to self‐manage after ED discharge (Hillier et al. 2019). To ensure safe and effective transitions of care, there is a critical need for healthcare professionals skilled in the care of older people (navigators) who can collaboratively initiate specialised assessments with a multidisciplinary team, identify barriers to discharge and support communication when transitioning from the ED to a primary care setting (Bambach and Southerland 2021; Dufour et al. 2020; Hillier et al. 2019).

Patient navigation, a process where a person (navigator) engages with the patient to determine barriers to accessing care and the health system and provides information to address those barriers, is established across various countries to improve access to primary care (Peart et al. 2018; Tang et al. 2021; Walkinshaw 2011). Patient navigators can have varied backgrounds and experiences, including mental health clinician, nursing staff, community health worker, social worker, clinician, nurse practitioners, pharmacist and case manager (Tang et al. 2021). They are trained to understand each patient's unique needs (Peart et al. 2018), and to help patients successfully navigate complicated systems in accessing the healthcare system (Ustjanauskas et al. 2016). ED navigators, in particular, support with patient flow and ensure people move through their ED journey as quickly as possible. Their roles may include but are not limited to assigning patients in room, ensuring completion of tests, following up on tests, communicating with doctors when the test results are ready, coordinating with consultations and community partners, identifying resources to support care and communicating with patient and families (Peretz et al. 2023). Despite the success of patient navigator interventions in cancer care (Atzema and Maclagan 2017; Hannah Budde et al. 2021; Ranaghan et al. 2016), evidence suggest mixed results regarding their effectiveness in facilitating transitions of older people from the ED to primary care. This creates ongoing challenges in recommending optimal ED care transition strategies to service users and their families. Considering policymakers' emphasis on support for ED navigator role implementation (Lowthian et al. 2015; Peart et al. 2018), the gaps in the comprehensive evaluation of the ED navigator interventions are quite surprising.

Often, measures of quality for complex care transitions to community focus on readmission rates and timely access to primary care follow‐up as direct measures of intervention success (Jeffs et al. 2013). Other measures such as examining patient‐centredness, equity and safety within the context of resource stewardship and financial impacts are often not included (Gettel et al. 2022; Institute of Medicine 2001; Peart et al. 2018). It is crucial to understand how ED navigator interventions are characterised and which outcome measures are employed to enhance the intervention and its evaluation. By doing so, we can facilitate the development of research studies that capture the multifaceted nature of quality as a cumulative socio‐cultural experience (Hanefeld et al. 2017).

Review studies on ED‐based transition interventions and measures have mostly focused on improving ED performance in general and/or listed number of studies focusing on ED patient navigators descriptively without discussing interventions and their effectiveness involving them (Austin et al. 2020; van den Broek et al. 2023; Atzema and Maclagan 2017; Lowthian et al. 2015). The limited research on interventions involving ED patient navigator hinders our ability to assess the effectiveness of their roles, potentially resulting in lower quality of care for patients, increased strain on the healthcare system and challenges in addressing these issues (Hanefeld et al. 2017). Our review will address this research gap.

### 1.1 Purpose

The purpose of this scoping review is to identify how ED patient navigator interventions for older people (≥ 65 years) transitioning to a primary care setting are described and evaluated in the literature.

## 2 Review Design and Methods

We followed the latest JBI guidance for the conduct of our scoping review (Peters et al. 2021). A scoping review is often used to understand complex questions such as ours and synthesise knowledge aimed at guiding practice, policy or programme development where available information is diverse and/or limited without providing guidance to clinical decision‐making (Arksey and O'Malley 2005; Peters et al. 2021; Schick‐Makaroff et al. 2016). The updated JBI framework is the up‐to‐date guidance for scoping reviews, which was developed via extensively reviewed literature, discussion with stakeholders and experts and feedback from the presentation at scientific conferences (Peters et al. 2021). It provides guidance on several methodological issues, analysis steps (extraction, synthesis) and presentation of results and sheds light on implications for practice and research. We followed the framework to a) formulate review objective; identify inclusion/exclusion criteria, study selection, data extraction and presentation, data analysis, results and evidence summary; conclusion and making recommendations. We used the Preferred Reporting Items for Systematic reviews and Meta‐analyses extension for Scoping Reviews (PRISMA‐ScR) Checklist to report the results of the scoping review (Tricco et al. 2018). The checklist can be found as Table S1. We did not conduct quality appraisal of the studies because our main purpose of the scoping review was to map the available evidence on ED navigator interventions and the measures used to monitor and examine them. If we were determining the effectiveness of the ED navigator interventions through a traditional systematic review, mixed‐method review, or something similar, then quality assessment would have been conducted and decisions made about inclusion and/or interpretation based on the methodological quality of the studies.

### 2.1 Search Strategy

Search filters available from the University of Alberta were combined with a search strategy developed with the guidance of a University of Alberta librarian. A search of three databases (Medline, EMBASE and CINAHL) was undertaken in July 2023. We did not include grey literature. We searched key terms such as ‘elderly adults’, ‘patient navigator’ and ‘emergency room’, selected using the *Population, Concept, Context* (PCC) framework (Peters et al. 2021). An example search strategy for Medline is provided in Table S2. All three databases were selected for their large repository of health sciences, peer‐reviewed content. CINAHL is known for its large volume of nursing‐focused literature, while EMBASE and Medline are considered the two largest databases of biomedical and life sciences journals (Nekolaichuk 2023). EMBASE also provides access to a wider variety of titles than Medline (Nekolaichuk 2023). No date filters were applied to this search as, to our knowledge, no previous reviews have been published looking to answer the question of identifying ED navigator interventions described and evaluated.

### 2.2 Study Selection

Articles were included if there was a distinct patient navigator role performed by a health care professional that facilitated the transition of care for older people to the community setting. Studies were excluded if the community disposition was to a care facility as this question was intended to focus on those older people who were living independently or using home‐based community services. Articles with either quantitative or qualitative outcomes were included. Any study where ED staff referred patients to a community treatment team for follow‐up was excluded if there was no ED‐based member of the community treatment team facilitating this intervention. Inclusion and exclusion criteria (Table 1) were refined during a consensus meeting after a screening exercise with a small subset of abstracts that were independently reviewed by two reviewers (KP, CP). Refining criteria clarified the focus to only older people transitioning to nonfacility living and focused on looking at studies with a designated navigator role, not a process done by existing ED staff. Two reviewers (KP, CP) screened titles and abstracts with consensus meetings held to resolve conflicts. A similar procedure was followed for full‐text screening. References and screening were managed using Rayyan.

**TABLE 1:** Inclusion and exclusion criteria.

Inclusion criteria	Exclusion criteria
Studies that include all of the following: Use a health care provider in the role of health system navigator (HSN), Focus on the transition from ED to the community without admission, Focus on interventions that occurred in an ED observation unit, Has a patient intervention performed by a navigator role	Studies that include any of the following: Use of HSN in Non‐ED settings (i.e., referral by ED staff to a community team, but there is no ED‐based intervention) Focus on education provided to staff or health navigator role. Inpatient admission within the ED Patients discharged to facility living (i.e., LTC, supportive living, etc.).
Studies that focused on Older adults (65 years old) or The mean age of participants was 65 years old or older.	Studies that include older adults but results specific to older adults are not reported.
All peer‐reviewed full‐text English language studies, including qualitative, quantitative, or mixed‐method designs.	Commentaries, thesis papers, reports, opinion articles, protocols, literature reviews, conference abstracts, grey literature, and studies unable to locate full text

### 2.3 Data Extraction and Analysis

We extracted study characteristics and substantive findings including: primary author(s), year of publication, country where the research was conducted data collection and analysis methods, summary description of interventions, outcome measures and main findings. Data extractions were completed by the primary reviewer (KP) and validated by two reviewers (RD, KP). Two research members (KP, KT) met during the research process to review and discuss extractions and preliminary findings.

Data were analysed using basic descriptive content analysis, as recommended by Peters et al. (Peters et al. 2021). Findings were collated and categorised based on distinct transition actions executed by ED navigators: assessment, consultation, liaison, developing a plan of care, referral and follow‐up. Outcome measures with similar conceptualisation and wording (i.e., ED discharge rate, hospital admission rate, length of stay, etc.) were grouped together and counted to understand frequency across the 10 studies. Outcome measures with unique wording and/or conceptualisation were counted as occurring once.

## 3 Results

**FIGURE 1:**
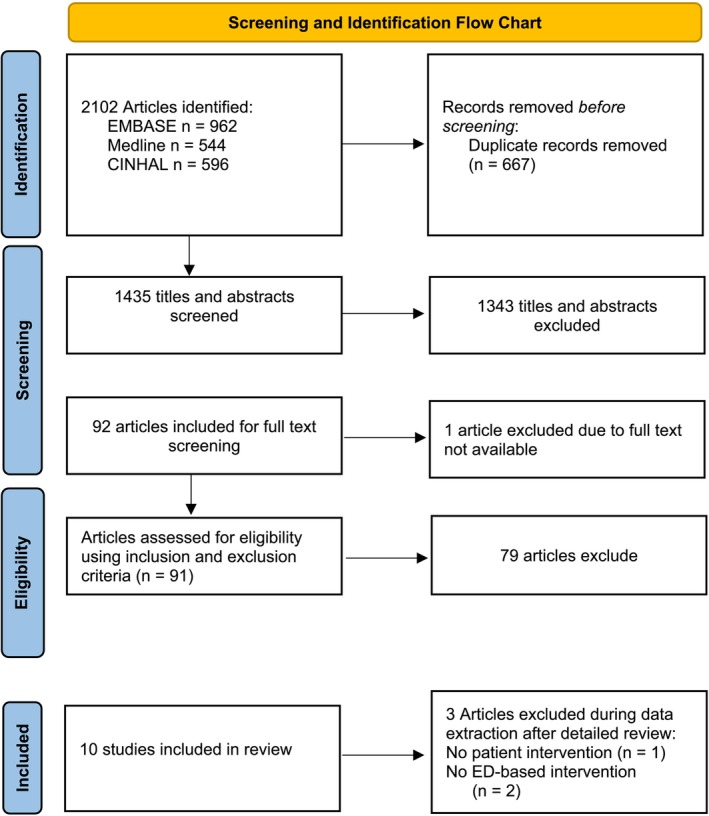
Preferred reporting items for systematic review and meta‐analyses (PRISMA) flow chart of literature search, screening, and selection process.

Out of 2102 articles, we included a total of 10 articles after duplicate removal and the screening process. Details of the screening and selection process are presented in the PRISMA‐SCR flow chart shown in Figure 1.

### 3.1 Study Characteristics

Of the ten studies, four were conducted in the United States (Aldeen et al. 2014; Gurley et al. 2020; Miller et al. 1996; Morse et al. 2019), two were conducted in both Australia (Basic and Conforti 2005; O'Grady et al. 1996) and the UK (Hardy et al. 2001; Poncia et al. 2000) and one study was conducted each in Canada (Guthrie et al. 2021) and Belgium (Heeren et al. 2019). All were single‐site studies. With the exception of one study (Morse et al. 2019), all used quantitative research designs. Quantitative studies used retrospective designs (*n* = 2), prospective cohort design (*n* = 2), quasi‐experimental before/after design (*n* = 2), multimethod design (*n* = 1), randomised control trial (*n* = 1) and nonrandomised controlled trial (*n* = 1). No study reported a theoretical framework used to guide study design, interventions implemented or outcomes measured. Study characteristics are presented in Table S3, and a detailed study‐level description of intervention, outcome measures and main findings are presented in Tables S4 and S5. Table 2 reports the number of studies representing intervention description and evaluation.

**TABLE 2:** Number of studies representing intervention description and evaluation.

Description of interventions	N studies
Intervention components	
Assessment	10
Liaison	4
Consultation	2
Develop plan of care	8
Referral	10
Follow‐up	7
Navigator skills/knowledge components	
Specialisation in gerontology	4
Training	6
Approaches used to provide care to patients during their transition	
Single provider	8
Team‐based approach	2
Outcome measures used to evaluate ED navigator interventions	
Death rate	2
Description of common patient concerns after ED discharge	1
Description of nursing responses after ED discharge	2
Description of type of follow‐up care received	1
Discharge rate	1
Disposition	1
ED length of stay	5
ED visits or re‐presentation within a specified time frame	4
Functional decline	2
Hospital admission or admission at re‐presentation rate	7
Hospital length of stay	6
Number of physician visits post ED discharge	1
Patient satisfaction of care provided	3
Rate of patient‐reported adherence to new medications	1
Rate of patient‐reported well‐being or quality of life	2
Change in living situation after ED discharge	2
Rate of dependency in basic ADLs and iADLs	1
Rate of new advanced care plans	1
Rates of problem resolution within a time frame	1
Rates of service utilisation	3

### 3.2 Description of Interventions

#### 3.2.1 Intervention Components

Descriptive analysis of ED navigator interventions revealed six (6) different components: assessment, consultation, liaison, development of care plan, referral and follow‐up (Table 2). In this review, ‘consultation' refers to the ED navigator accessing in‐hospital services to assess care needs and facilitate community discharge. The term ‘referral’ describes the ED navigator using community‐based services to facilitate further assessment after discharge and/or follow‐up care. Two studies included both access to consultation and referral components (Aldeen et al. 2014; Hardy et al. 2001) while the others utilised either outpatient referral workflows to community service providers or consultation to in‐hospital services (Basic and Conforti 2005; Gurley et al. 2020; Guttman et al. 2004; Heeren et al. 2019; Miller et al. 1996; Morse et al. 2019; O'Grady et al. 1996; Poitras et al. 2020). Two studies involved a nurse providing telephone follow‐up post ED discharge, focusing on common themes during follow‐up care and measuring participants' connection to community services (Morse et al. 2019; Poitras et al. 2020).

Most studies (90%) included at least three intervention components, with half of the studies incorporating five out of the six identified components (Aldeen et al. 2014; Guttman et al. 2004; Hardy et al. 2001; Miller et al. 1996; O'Grady et al. 1996). None of the studies included all six components of assessment, consultation, liaison, developing a plan of care, referral and follow‐up. Studies that combined more than 4 intervention types reported more positive and significant outcomes (Aldeen et al. 2014; Guttman et al. 2004; Hardy et al. 2001; Miller et al. 1996; O'Grady et al. 1996). However, studies using 4 or fewer components of interventions reported negative or nonsignificant effects on outcomes (Basic and Conforti 2005; Heeren et al. 2019).

#### 3.2.2 Navigator Skills/Knowledge Components and Training

In included studies, ED navigators were either specialised in providing care to older people or were provided additional training as needed for their role. Four studies used nurses specialised in gerontology to administer assessments and facilitate interventions (Basic and Conforti 2005; O'Grady et al. 1996; Guttman et al. 2004; Morse et al. 2019). Five of the studies reported providing an ED or community nurse with additional training (Aldeen et al. 2014; O'Grady et al. 1996; Guttman et al. 2004; Hardy et al. 2001; Miller et al. 1996). Three studies did not identify the amount of time spent on training (Hardy et al. 2001; Miller et al. 1996; O'Grady et al. 1996). Of the two that did, one invested in an extensive 4‐month comprehensive multidisciplinary training programme with geriatric specialists (Aldeen et al. 2014) while the other provided a week of intensive gerontology orientation (Guttman et al. 2004). In two studies, training was narrowly focused on how to administer a comprehensive assessment to identify unmet needs of older people that would benefit from the study's intervention (Hardy et al. 2001; Miller et al. 1996). One study described a just‐in‐time training approach which provided extra support to the community‐based health care team when knowledge gaps were identified (O'Grady et al. 1996). Two studies did not report providing additional training to the health care professional in the navigator role (Gurley et al. 2020; Poncia et al. 2000). All but one study used nurses to facilitate interventions; The one study that did not used physiotherapists to facilitate determination of disposition based on functional needs, not necessarily medical ones (Poncia et al. 2000).

#### 3.2.3 Approaches Used to Provide Care to Patients During Their Transition

Two studies utilised a team‐based approach, which involved an ED‐based navigator working in conjunction with a designated community follow‐up team consisting of community nurses and personal care aids. This approach aimed to provide continuous care to patients until they could be transitioned to community services or no longer required support. Using this model, both studies reported qualitative and statistically significant quantitative improvements in communication and coordination of ongoing care needs and outstanding community referrals. This enhanced team's ability to dynamically meet patient needs and redistribute workloads when necessary (Hardy et al. 2001; O'Grady et al. 1996). The remaining eight studies focused on interventions involving a single healthcare professional, usually a nurse with ED experience in the ED. These interventions utilised referral and/or consultation to link patients with the necessary assessments and follow‐up care (Basic and Conforti 2005; Gurley et al. 2020; Guttman et al., Guttman et al. 2004; Heeren et al. 2019; Miller et al. 1996; Morse et al. 2019; O'Grady et al. 1996).

### 3.3 Outcome Measures Used to Evaluate ED Navigator Interventions

Descriptive content analysis revealed that 20 distinct outcome measures were employed in the literature to describe and evaluate ED navigator interventions (Table 2). The three most frequently utilised outcome measures were hospital admission rates, number of ED visits, and length of stay, which are elaborated below.

#### 3.3.1 Hospital Admission and Readmission

Hospital admission and/or readmission was the most frequently observed outcome measure (*n* = 6). In studies using prospective cohort designs, implementation of ED navigator intervention decreased admissions (*n* = 1) or had no statistically significant impact on the outcome (*n* = 1). Authors in one of the studies highlighted that patients receiving interventions were more likely to be admitted and suggested that this could be attributed to the more thorough assessment revealing subtle geriatric syndromes that might have been overlooked by busy ED staff (Aldeen et al. 2014). Studies using quasi before and after designs report an increase in hospital admission rates (*n* = 1) or nonsignificant findings (*n* = 1). One study using quasi design reported that the likelihood of selection bias towards sicker patients posed challenges in comparing cohorts and resulted in no statistical difference in admission rates (Guttman et al. 2004). In a multimethod study, a decrease in admissions was observed and a randomised controlled trial study reported nonsignificant findings.

#### 3.3.2 Emergency Department Presentations

Four studies reported ED visits or revisit outcomes. One quasi‐experimental study comparing ED visits between cohorts at 30 and 90 days showed similar rates of readmission (Heeren et al. 2019); while another quasi‐before–after study reported that patients who received an ED navigator intervention were less likely to have a repeated ED visit (Guttman et al. 2004); however, the findings did not reach statistical significance. In a prospective cohort study, ED re‐presentation at 3 days was assessed, and it was identified that while a reduction in admissions was observed due to increased discharges, the rate of ED visits at 3 days remained unaffected (Aldeen et al. 2014). Although a nonsignificant finding was observed, authors in a nonrandomised controlled trial reported that patients who received an ED navigator intervention, and findings suggested decreased ED visits (Miller et al. 1996).

#### 3.3.3 Length of Stay

The duration of ED stays and hospitalisations were the third most common category of outcome measures identified in the studies (*n* = 8). Prospective cohort studies reported prolonged ED stays but a shorter hospital stay (*n* = 1), or reduced hospitalisation time (*n* = 1) in intervention groups. A study using retrospective cohort design reported longer ED length of stay (Gurley et al. 2020), and a nonrandomised controlled trial reported longer ED length of stay and reduced hospitalisation time in the intervention group (Miller et al. 1996). There was a decrease in length of ED stay in intervention groups in a quasi‐pre–post design study, but no difference in hospital length of stay was observed (Heeren et al. 2019). In a randomised controlled trial, no change in ED and hospital length of stay was observed (Basic and Conforti 2005).

## 4 Discussion

To our knowledge, there is no existing review that describes ED navigator interventions for older people and the outcome measures used, making this review a unique contribution to the literature. Based on the studies reviewed, it is clear that there is still a gap regarding the effectiveness of interventions to facilitate older persons' care transitions, as most studies lack consistency in how interventions were designed and which components were included to facilitate care transitions for older people from the ED to the community. The most common unique outcome measures used to evaluate interventions were rates of hospitalisation/readmission, the number of ED presentations, and ED/hospital length of stay. Patient‐centred measures aimed at evaluating how well care is tailored to individual needs, such as patient satisfaction with the care services provided and perception of their own health, were often only seen within a single study (Institute of Medicine 2001). Although there are similarities across ED settings, more consistency between studies regarding intervention design and outcome selection would be helpful in evaluating and comparing health system quality and performance. Further research into understanding which patients would likely benefit from an ED navigator intervention could be an important focus for future studies to guide the tailoring and contextualisation of interventions across ED settings and the care continuum. We also observed that most studies were based on quantitative designs, revealing a significant gap in the in‐depth understanding of the ED navigator role using qualitative designs.

### 4.1 Interventions and Outcome Measures

This scoping review highlights the wide variation in the definition of an ED navigator role and the lack of consistency in core intervention components across studies (Jessup et al. 2018). The complexity of interventions also varied, with the number of included components ranging from 2 to 5, and no study including all 6 identified components across the 10 studies (Table 2). Studies that had interventions comprised of 4 or more components showed reduced repeated ED visits and shorter hospital admissions, indicating that multifaceted interventions could be a promising area for future research. A broader role for ED navigators may provide them with the capacity, mandate and resources to address individual patient needs more comprehensively and in a timely manner.

There are still gaps in understanding which intervention components are crucial to include, or how context impacts intervention implementation and effectiveness, hindering our ability to definitively identify key strategies for transitioning patients from the ED to community care (Lowthian et al. 2015). For example, a systematic review of patient navigation for cancer care across the continuum found strong evidence that navigation interventions are effective in areas such as early cancer detection, screening and attending follow‐up care, yet found inconclusive findings regarding the effectiveness of patient navigation for ensuring treatment compliance (Chan et al. 2023). This may be because of differences in care needs during different phases of care, or differences in external or contextual factors that impact patient ability to adhere to treatment. For example, whether or not a consult or referral is followed through with may be dependent on an older person's ability to attend outpatient appointments in a community which requires transportation they may not have access to on their own. Valtorta et al. associated weaker social connections in older people with increased admission rates and longer hospital stays, highlighting that a patient's social life can impact their health journey and is not easily addressed with an ED navigator intervention (Valtorta et al. 2018). Intervention components such as referral, consultation, and liaison connected older people to experts across care settings and may be important intervention components that require research to enhance their feasibility and effective implementation (Bowen et al. 2009) and to examine their efficacy in pragmatic trials (Patsopoulos 2011). Moreover, with an aging Canadian population, further research focused on identifying which patients would benefit most from ED navigation would be an important next step in enhancing intervention development and evaluation (Government of Canada 2022). Realist approaches may be warranted to examine what works for whom in particular contexts and through what mechanisms (Jack 2022).

There is a predominant focus on assessing the success of intervention by analysing admission rates, rates of ED visits and lengths of stay. This approach may limit our understanding of the effectiveness of ED navigator interventions. Other important metrics, such as patient satisfaction, service utilisation and patient function, are often overlooked. Studies evaluating outcomes in different healthcare settings could provide a more comprehensive view of intervention impact, especially in the context of care transitions. Research has shown that while ED navigator interventions may increase ED length of stay, they can lead to shorter hospital stays for admitted patients. Incorporating additional measures that assess the impact of the interventions across various care settings (e.g., ED, inpatient units, short‐stay facilities and community care) could enhance the evaluation of quality and performance. A systematic review and Delphi process on quality indicators for older people throughout the care continuum emphasise the importance of using a set of measures that span different care settings to evaluate healthcare delivery effectively for meaningful system enhancements (Tate et al. 2022). Its inclusion of grey literature can also be used in conjunction with this review to identify measures that are promising related to this topic area but require rigorous evaluation using established methods. We report commonly used outcome measures and comparison of the number of components and outcome measures in Supplemental files 4 and 5.

### 4.2 Implications for Research and Practice

A common reason for implementing an ED navigator is to reduce the costs of acute care services by connecting patients with timely follow‐up in the community or facilitating more urgent assessments to avoid costly hospital visits. With ED navigators and health administrators often being nurses, there is an opportunity for professional ownership in understanding how to best execute the process. While focusing on a single measure can be useful when evaluating intervention effectiveness (Austin et al. 2020), using multiple measures can help uncover contextual issues that may be obscured by narrowly focused observations. For example, ED re‐presentation can be due to new unrelated medical issues, the need for reassurance, or the progression of a disease that was not always predictable (van Loon‐van Gaalen et al. 2023). A study examining the feasibility and relevance of quality indicators used in evaluating care transitions for older people found concerns about the appropriateness of chosen indicators and major gaps in capturing contextual information, especially across care settings (Tate et al. 2022). The dual impact of interventions, such as longer ED stays but shorter admission stays, may be overlooked when only measuring outcomes in a single setting (e.g., ED) rather than across care settings (i.e., from ED to community or hospital). This can result in an inaccurate representation of the true impact of the ED navigator role due to the complex nature of ED patients requiring a team‐based approach to care and follow‐up (Cadogan et al. 2016; Evans et al. 2009). Future research should focus on identifying outcome measures that can contextualise intervention effectiveness across care settings and provide a more nuanced understanding of how care transitions are either hindered or improved.

The evolution of healthcare has led to a longer living population that is more likely to experience higher levels of frailty and complex chronic health conditions. This increased complexity in care necessitates a skilled team of general and expert clinicians working collaboratively, which is often not readily available within an ED setting (Kojima et al. 2019). Historically, Canada has underinvested in community health services compared to hospital spending, resulting in reduced access for patients to regular preventative care, early community screening and treatment to manage health issues before they escalate (Canadian Institute for Health Information 2022; C.D. Howe Institute 2021). Consequently, patients often resort to using EDs to address their health needs, waiting until a crisis event occurs (Ly et al. 2021). Current trends in ED utilisation in Canada indicate that patient needs are not adequately met elsewhere in the healthcare system, or patients are unsure of how to access community services, leading to a shift in the role of EDs to provide more support in facilitating transitions in care (Canadian Institute for Health Information 2014). This is in addition to the traditional ED mandate of streamlined urgent or emergent management of life‐ or limb‐threatening illnesses. The successful creation and implementation of ED navigator roles may depend on a healthcare system that is focused on a sustainable care continuum rather than episodic care (Kojima et al. 2019).

## 5 Limitations

There are several limitations to this scoping review. Firstly, we restricted the search to peer‐reviewed studies published in online databases, potentially leading to publication bias. The aim of our scoping review was to identify what is published in academic literature on ED navigator interventions, meaning internal sources from health authorities and/or quality assurance agencies and institutions are not included in this paper. This was also necessary due to limited time and resources. The inclusion of only articles published in the English language may have resulted in missing important non‐English articles. Although evaluating the reference lists of existing reviews and selected studies could have identified more articles, this was not feasible due to resource and time constraints. We recognise that including three databases can be limiting in a scoping review, but due to limited resources in a graduate student project and given the optimal selection of databases, we believe this search to be sufficiently comprehensive due to the size and theoretical relevance of the included databases (Bramer et al. 2017; Ewald et al. 2022).

On the other hand, this study's strengths lie in the rigorous screening procedures carried out by independent reviewers and the research team's composition of methodological and content experts.

## 6 Conclusion

This scoping review contributes to the literature on transitions from the ED to primary care by examining how ED patient navigator interventions for older people transitioning to a primary care are described and evaluated. The review revealed inconsistencies in the number of components included in ED navigator interventions, reflecting the evolving role of ED navigators. Common outcome measures focus on admission rates, ED visits and length of stay, rather than evaluating the impact of interventions on care transitions across health care settings. Future research should determine essential activities for ED navigator interventions, identify patients who may benefit from navigation and track core outcomes across care settings. Patient‐centred outcome measures, such as patients' perception of their health needs being met, should be consistently included to assess intervention success.

## Author Contributions

K.P., C.P. and K.T. were involved in the conception and design of this study. With support from C.P. and K.T., K.P. ran searches in various databases. K.P. and C.P. conducted screening, data analysis and results reporting. R.D. assisted K.P. with data extraction, interpretation and table preparation. K.T. drafted the manuscript, and all authors revised and reviewed it as necessary. All authors confirm responsibility for this study.

## Conflicts of Interest

The authors declare no conflicts of interest.

## Supporting information


Data S1.

## Data Availability

Data sharing is not applicable to this study because we did not create or analyze any new data.
