# Supplementary material for: Emergency Department Navigator Interventions and Outcome Measures: A Scoping Review
Source: Int J Older People Nurs. 2025 Mar 31;20(3):e70026. doi: 10.1111/opn.70026 (PMC11959214; doi:10.1111/opn.70026)
Supplement: Supplementary file 1 — Data S1. [file OPN-20-e70026-s001.docx]

**Supplementary table 1:** Preferred Reporting Items for Systematic reviews and Meta-Analyses extension for Scoping Reviews (PRISMA-ScR) Checklist

| **SECTION** | **ITEM** | **PRISMA-ScR CHECKLIST ITEM** | **REPORTED ON PAGE #** |
| --- | --- | --- | --- |
| **TITLE** | | | |
| Title | 1 | Identify the report as a scoping review. | Title page |
| **ABSTRACT** | | | |
| Structured summary | 2 | Provide a structured summary that includes (as applicable): background, objectives, eligibility criteria, sources of evidence, charting methods, results, and conclusions that relate to the review questions and objectives. | 12 |
| **INTRODUCTION** | | | |
| Rationale | 3 | Describe the rationale for the review in the context of what is already known. Explain why the review questions/objectives lend themselves to a scoping review approach. | 15-17 |
| Objectives | 4 | Provide an explicit statement of the questions and objectives being addressed with reference to their key elements (e.g., population or participants, concepts, and context) or other relevant key elements used to conceptualize the review questions and/or objectives. | 17 |
| **METHODS** | | | |
| Protocol and registration | 5 | Indicate whether a review protocol exists; state if and where it can be accessed (e.g., a Web address); and if available, provide registration information, including the registration number. | Not applicable |
| Eligibility criteria | 6 | Specify characteristics of the sources of evidence used as eligibility criteria (e.g., years considered, language, and publication status), and provide a rationale. | 19 |
| Information sources* | 7 | Describe all information sources in the search (e.g., databases with dates of coverage and contact with authors to identify additional sources), as well as the date the most recent search was executed. | 19 |
| Search | 8 | Present the full electronic search strategy for at least 1 database, including any limits used, such that it could be repeated. | Supplementary table 2 |
| Selection of sources of evidence† | 9 | State the process for selecting sources of evidence (i.e., screening and eligibility) included in the scoping review. | 19-20 |
| Data charting process‡ | 10 | Describe the methods of charting data from the included sources of evidence (e.g., calibrated forms or forms that have been tested by the team before their use, and whether data charting was done independently or in duplicate) and any processes for obtaining and confirming data from investigators. | 20 |
| Data items | 11 | List and define all variables for which data were sought and any assumptions and simplifications made. | 20 |
| Critical appraisal of individual sources of evidence§ | 12 | If done, provide a rationale for conducting a critical appraisal of included sources of evidence; describe the methods used and how this information was used in any data synthesis (if appropriate). | Not applicable |
| Synthesis of results | 13 | Describe the methods of handling and summarizing the data that were charted. | 20 |
| **RESULTS** | | | |
| Selection of sources of evidence | 14 | Give numbers of sources of evidence screened, assessed for eligibility, and included in the review, with reasons for exclusions at each stage, ideally using a flow diagram. | 21, Figure 1 |
| Characteristics of sources of evidence | 15 | For each source of evidence, present characteristics for which data were charted and provide the citations. | 21, supplementary table 3 |
| Critical appraisal within sources of evidence | 16 | If done, present data on critical appraisal of included sources of evidence (see item 12). | Not applicable |
| Results of individual sources of evidence | 17 | For each included source of evidence, present the relevant data that were charted that relate to the review questions and objectives. | Supplementary table 4 &5 |
| Synthesis of results | 18 | Summarize and/or present the charting results as they relate to the review questions and objectives. | 21-26 |
| **DISCUSSION** | | | |
| Summary of evidence | 19 | Summarize the main results (including an overview of concepts, themes, and types of evidence available), link to the review questions and objectives, and consider the relevance to key groups. | 26-31 |
| Limitations | 20 | Discuss the limitations of the scoping review process. | 31 |
| Conclusions | 21 | Provide a general interpretation of the results with respect to the review questions and objectives, as well as potential implications and/or next steps. | 31-32 |
| **FUNDING** | | | |
| Funding | 22 | Describe sources of funding for the included sources of evidence, as well as sources of funding for the scoping review. Describe the role of the funders of the scoping review. | 32 |

JBI = Joanna Briggs Institute; PRISMA-ScR = Preferred Reporting Items for Systematic reviews and Meta-Analyses extension for Scoping Reviews.

* Where *sources of evidence* (see second footnote) are compiled from, such as bibliographic databases, social media platforms, and Web sites.

† A more inclusive/heterogeneous term used to account for the different types of evidence or data sources (e.g., quantitative and/or qualitative research, expert opinion, and policy documents) that may be eligible in a scoping review as opposed to only studies. This is not to be confused with *information sources* (see first footnote).

‡ The frameworks by Arksey and O’Malley (6) and Levac and colleagues (7) and the JBI guidance (4, 5) refer to the process of data extraction in a scoping review as data charting*.*

§ The process of systematically examining research evidence to assess its validity, results, and relevance before using it to inform a decision. This term is used for items 12 and 19 instead of "risk of bias" (which is more applicable to systematic reviews of interventions) to include and acknowledge the various sources of evidence that may be used in a scoping review (e.g., quantitative and/or qualitative research, expert opinion, and policy document).

*From:* Tricco AC, Lillie E, Zarin W, O'Brien KK, Colquhoun H, Levac D, et al. PRISMA Extension for Scoping Reviews (PRISMAScR): Checklist and Explanation. Ann Intern Med. 2018;169:467–473. [doi: 10.7326/M18-0850](http://annals.org/aim/fullarticle/2700389/prisma-extension-scoping-reviews-prisma-scr-checklist-explanation).

## Supplementary table 2: Medline Database Search Strategy

| **PCC** | **Search Terms** |
| --- | --- |
| Population-Elderly Adults | Aged/ or Health Services for the Aged/ or Senior Centers/ or (elders or elderly or geriatric* or gerontolog* or  "old age" or  "senior citizen*" or  (seniors not "high school") or ((older or mature) adj3 (adult* or person* or people or patient or patients or man or men or woman or women)) or centenarian* or nonagenarian* or octogenarian* or septuagenarian* or sexagenarian* or dottering or decrepit or tottering or overaged or "oldest old" or supercentenarian*) or elder* or geriatric* or senior* or "old* adult*" or "old* individual*" or "old* person*" or "old* people" or gerontolog*.mp. |
| Concept-Patient Navigator | (navigator* or "case manage*" or "transition coordinator" or "transition co-ordinator*" or liais* or "discharge* coordinator*" or "discharge* co-ordinator*" or "home coordinator*").mp. |
| Context: Emergency Room | Emergency Treatment/ or Emergency Medicine/ or emergency medical services/ or emergency service, hospital/ or trauma centers/ or triage/ or exp Evidence-Based Emergency Medicine/ or exp Emergency Nursing/ or Emergencies/ or emergicent*.mp. or casualty department*.mp. or ((emergenc* or ED) adj1 (room* or accident or ward or wards or unit or units or department* or physician* or doctor* or nurs* or treatment* or visit*)).mp. or (triage or critical care or (trauma adj1 (cent* or care))).mp. or "Emergency room*".mp. or "emergency department*".mp. or "trauma centre*".mp. or "trauma center*".mp. or "accident and emergency".mp. or "critical care".mp. or "urgent care".mp. |

## Supplementary table 3: Characteristics of Included Studies

| **S.No.** | **Author(s), Year, Journal, Country** | **Theoretical Framework** | **Research Design** | **Purpose** | **Sample & Setting** | **Data Collection Method & Analysis** |
| --- | --- | --- | --- | --- | --- | --- |
| 1. 1. | Aldeen, A.Z., et al., 2014  Journal of the American Geriatrics Society  US | Not reported | Prospective cohort design | To train Geriatric Emergency Department Innovations (GEDI) ED nurse liaisons, describe characteristics of patients assessed by GEDI nurse, and measure the admission rate of these patients. | N=7,214 ≥ 65 years of age (GEDI=408, Non-GEDI=6,806)  Emergency department (n=1) | Data collection method:  Patient record review  Analysis:  Descriptive,  T-test, Wilcoxon rank-sum test |
| 1. 2. | Basic, D & Conforti, D. 2005  Australian Health Review  Australia | Not reported | Prospective randomized controlled trial | To evaluate the effect of a nurse experienced in multidimensional assessment and care of the elderly in reducing admissions, functional decline in hospital or length of stay. | N= 224 older adults presenting to ED who were not severely ill (Intervention group [I]: 18 with functional decline, 61 with no functional decline; control group [C]: 60 with functional decline, 21 with no functional decline)  Emergency department (n=1) | Data collection method: Patient record review  Analysis:  Descriptive  Multivariate logistic regression  Multivariate Cox proportional hazards (for length of stay) |
| 1. 3 | Grady et al., 1996  Australian Health Review  Australia | Not reported | Multimethod design (Retrospective cohort study, outcome study, interview survey, and action research approach) | To determine whether the hospital admission of some older patients was  Avoidable  To establish an optimal follow-up care protocol for elderly patients presenting  to and discharged from the emergency department. | N=60 older adults N= 56 corresponding general practitioners  N=38 carers of the patients  Emergency department (n=1) | Data collection method: Patient record audit  Survey & structured interview with patients and care givers.  Reflective individual and group discussion sessions with clinicians, and care staff.  Analysis:  Multiple logistic regression  Incorporation of results (discussion, and interviews) into the implementation process to form the continuous feedback loops |
| 1. 4 | Gurley et al., 2019  Injury Prevention  US | Not reported | Retrospective observational design | To demonstrate that the novel clinical pathway is safe, viable, and reduces hospital admission in this cohort of patients. | N=1296 patient  Emergency department (n=1) | Data collection method:  Patient record review  Analysis:  Descriptive  Chi-square test for categorical variables to make comparisons across disposition category  Kruskal-Wallis test to compare ED length across disposition categories |
| 1. 5 | Guttman et al., 2004  Academy Emergency Medicine  Canada | Not reported | Prospective pre/post design | To compare usual discharge care (pre;  control) with that of an ED-based discharge planning program (post; intervention) for elder patients discharged from the ED | N= 1724 (Control [C]: 905; Intervention[I]: 819) 75 years and older who were discharged from the ED  Emergency Department (n=1) | Data collection method:  Administrative database review maintained by researchers.  Semi-structured patient interviews.  Patient record review.  Analysis:  Multivariate cox proportional hazards regression |
| 1. 6 | Hardy et al., 2001  Emergency Medicine Journal  UK | Not reported | Prospective cohort design | To validate an accident and  emergency (A&E) based approach to assisting early discharge or avoiding admission to acute hospital beds by means of two separate teams, one in the hospital and the other in the community, working closely together at the interface between primary and secondary health care. | N= 788 (Intervention group [I]: 257; historical control group [C]: 531). Only 149 patients in the intervention group were comparable to a historical control group  Emergency Department (n=1) | Data collection method:  Administrative data review  Patient satisfaction survey  Analysis:  Descriptive  Cost estimation |
| 1. 7 | Heeren et al., 2019  BMC Geriatrics  Belgium | Not reported | Single center, quasi-experimental before-after study design | To evaluate the effectiveness of the URGENT care model compared to usual care on the unplanned ED readmission rate, as primary outcome, and secondary outcomes (i.e. ED length of  stay (LOS), hospitalization rate, in-hospital LOS, higher  level of care, functional decline and post-hospitalization  mortality) of community-dwelling, older adults. | N= 1680 (Intervention [I]: 886; Control [C]: 794) patients aged 70 years or older  Emergency Department (n=1) | Data collection methods:  Patient record review  Semi-structured patient interviews.  Analysis:  Descriptive  Multivariable logistic regression  Cox proportional hazard regression models  Lognormal model to compare the ED length of stay and the in-hospital length of stay |
| 1. 8 | Miller et al., 1996  Journal of the American Geriatrics Society  US | Not reported | Nonrandomized controlled trial | To evaluate the effects of case finding and liaison service program for older patients visiting the emergency department | N= 770 (Intervention[I]: 385, Control[C]: 385) 65 years and older  Emergency Department (n=1) | Data collection method:  Patient record review  Semi-structured interviews  Analysis:  Cost estimate  Chi-square and t-test |
| 1. 9 | Morse et al., 2019  Geriatrics  US | Not reported | Retrospective chart review with qualitative analysis | To characterize the content and interventions performed during follow-up phone calls made to patients discharged from the Geriatrics Emergency Department Innovation (GEDI) Program and to demonstrate the benefit of these calls in the care of older adults discharged from the emergency department (ED). | N=57 adults aged 65 years and older  Emergency Department (n=1) | Data collection method:  Patient chart review  Semi-structured patient interviews  Analysis:  Descriptive    Content and constant comparative techniques to assess the responses and create a list of major themes |
| 1. 10 | Poncia et al., 2000  Journal of Accident and Emergency Medicine  UK | Not reported | Retrospective cohort study design | To assess the needs of these patients the day after discharge, target patients for appropriate interventions and identify critical incidents. | N=551 patients aged 75 years and older  Accident and Emergency (A&E) Department (n=1) | Data collection method:  Patient record review  Telephone follow-up using a Semi-structured questionnaire  Analysis:  Descriptive |

**Supplemental table 4**: Overview of intervention & outcomes measured

| Author(s), Year | Team approach or single provider | Ability to access in-hospital consultations, outpatient referral, both or none | Intervention Components | Number of Outcomes Measured |
| --- | --- | --- | --- | --- |
| Aldeen, A.Z., et al., 2014 | Single nurse | Both | Assessment  Consultation  Develop Plan of Care  Referral  Follow-up | 7 |
| Basic, D & Conforti, D. 2005 | Single nurse | Outpatient referral | Assessment  Liaison  Develop Plan of Care  Referral | 3 |
| Grady et al., 1996 | Single nurse with community care team | Outpatient referral | Assessment  Liaison  Develop Plan of Care  Referral  Follow-up | 5 |
| Gurley et al., 2019 | Single physiotherapist | Outpatient referral | Assessment  Develop Plan of Care  Referral | 4 |
| Guttman et al., 2004 | Single nurse | Outpatient referral | Assessment  Liaison  Develop Plan of Care  Referral  Follow-up | 5 |
| Hardy et al., 2001 | Nurse liaison and clinical assistant with community care team | Both  Consultation only with OT/PT | Assessment  Consultation  Develop Plan of Care  Referral  Follow-up | 3 |
| Heeren et al., 2019 | Single liaison nurse | Outpatient referral | Assessment  Referral | 7 |
| Miller et al., 1996 | Single liaison nurse | Outpatient referral | Assessment  Liaison  Develop Plan of Care  Referral  Follow-up | 9 |
| Morse et al., 2019 | Single liaison nurse | Outpatient referral | Assessment  Develop Plan of Care  Referral  Follow-up | 2 |
| Poncia et al., 2000 | Single liaison nurse | Outpatient referral | Assessment  Referral  Follow-up | 2 |

## Supplemental table 5: Detailed description of interventions, outcome measures, & main findings

| **S.No.** | **Author(s), Year, Journal, Country** | **Description of Interventions** | **Outcome Measures** | **Main findings** |
| --- | --- | --- | --- | --- |
| 1. 1. | Aldeen, A.Z., et al., 2014  Journal of the American Geriatrics Society  US | *Intervention*: GEDI model   - 4 ED nurses underwent 4 months of multi-disciplinary training. - Older adults (≥ 65) in the ED were screened for GEDI program eligibility using the Identification of Seniors at Risk (ISAR) tool. - GEDI nurse used standardized tools to assess cognition, delirium, fall risk, functional status, care transitions, and caregiver strain. - GEDI nurse facilitated multidisciplinary consultation, as needed. - GEDI nurse coordinated and discussed plan of care with primary ED team and primary care provider, respectively. - Older adults were discharged home with an appropriate plan. - Phone follow-up at 1-3 days and 10-14 days. | Discharge rate by ESI (acuity) score  Admission rate based on ESI (acuity) score  Death rate  30-day inpatient readmission rate  3-day ED revisit  ED length of stay  Inpatient length of stays | - GEDI patients were more likely to be older than non-GEDI, be on Medicare, and have an inpatient admission in the last 30 days. - There were 13% fewer admissions overall and 16% fewer in those with an Emergency Severity Index (ESI) score of 2. - The reduction in admission was due to more discharges, and the increase in discharges did not occur at the expense of a higher 3-day ED revisit rate. - Death rate not included in discussion; patients who died were excluded. - ED length of stay for GEDI patients was 1.1hrs longer than the control group. - GEDI patients who were admitted had a shorter inpatient stay.   Limitations:   - Staff resources limited the number of patients accepted. - Possible selection bias with GEDI-intervention patients resulting in higher admission rates, but short hospitalizations. |
| 1. 2. | Basic, D & Conforti, D. 2005  Australian Health Review  Australia | *Intervention*: Early geriatric assessment in the form of Aged Care Nurse (ACN) intervention.   - Patients randomized to intervention and control group after detailed baseline assessment by ACN. - Baseline assessments of intervention group were recorded in the medical file, with an emphasis on active geriatric problems. - ACN liaised with the patients’ carers and health care providers, including general practitioners and community-based agencies - Patients discharged home from the ED with unmet medical, functional, psychological or social needs were referred to a community or social agency and/or the general practitioner (GP). - CAN documented suggestions in the medical file, including recommendations for formal geriatric assessment for patients who were admitted. - Baseline assessment data form control group were withheld, and the nurse had no further involvement in their care (including out-of-hospital care). - Inpatient referral rates to geriatricians, physiotherapists, occupational therapists, and social workers in both the control and intervention groups were measured. | Admission rate to hospital  Length of stay in hospital  Rate of functional decline | - The intervention had no significant effects on hospital admission between the two groups or LOS or functional decline during hospitalization. - 15% referred for geriatric medical review, 67% referred to a physiotherapist 57% to a social worker 47% to an occupational therapist and 18% were not referred   Limitations:   - Findings cannot be applied to medically unstable patients. - Unable to determine if non-intervention patients were receiving similar care due to staff observations of the study interventions. - Multiple challenges in using functional decline as an outcome measure. |
| 1. 3 | Grady et al., 1996  Australian Health Review  Australia | *Intervention*: Best practice project (Quick response program (QRP), and extended hours community health nursing service)   - Project overseen by management committee consisting of representatives’ form unions, staff (clinical and management) and key stakeholders - Four project teams developed various aspects of the project (QRP operation project team, skills/practice and education project team, general practitioner and community liaison project team, and evaluation and research project team) - QRP liaison nurse (a community health nurse with gerontological training) was based in the ED from 8:30 am to 7:00 pm, 7 days per week bridging between the patient and the system advocating for the best outcome. - She assessed patients, determined their eligibility, liaised with ED staff and GP, developed plans and notified the service providers. - QRP arranged to transport the patient home and provided intensive short-term community health nursing and allied health services within a few hours of the patient leaving ED or referral - At 5 days a decision was made by the case coordinator at that time, in consultation with GP, whether to continue with QRP for a few more days discharge to self-care, or to regular community health/support services, or consider other options. - During the last six months, GPs could directly access QRP in the community after an assessment to determine if QRP could meet the patient’s needs. - Education strategies were implemented to strengthen community health nurses’ aged care assessment skills. - Allied health services were contracted from hospital-based physiotherapists, occupational therapists and social workers. Urgent cases were seen in the ED. Non-urgent cases were referred to the regular community allied health services for ongoing treatment. | ED length of stay  Rate of hospitalization  Rate of presenting problem resolution/improvement within 60days.  Rates of community services utilization pre and post intervention.  Patient satisfaction with QRP services. | - ED waiting time and length of stay not affected by the intervention. - One-third of those on the QRP likely avoided admission to the ward. Of those readmitted to hospital, 3% were considered a delayed admission. - The greatest predictors of those patients requiring treatment under the QRP were increasing age, arrival by ambulance, and a major diagnostic category (MDC) of injury or respiratory, circulatory, digestive, urological or musculoskeletal illness - Pre-intervention, two-thirds of QRP patients were not receiving community service at the time of their ED visit. Post-intervention, two thirds of QRP patients were discharged to regular community health or support services. - 85% patients satisfied with QRP service   Limitations:  None explicitly identified |
| 1. 4 | Gurley et al., 2019  Injury Prevention  US | *Intervention*: ED based physical therapist consult coupled with pre-existing ED case management   - A physical therapist ED consult service was available from 8am-5pm 7 days a week. ED consults were prioritized and these consults were placed electronically. - After the patient was medically cleared for discharged, a bedside ambulatory trial was performed by the ED nurse. - Patients unable to complete the trial received a physical therapist and case management consultation after approval from the medical team (primary and secondary resident and attending physician). - Patients with an anticipated prolonged ED length of stay placed in observation status and were clinically re-evaluated by attending physician every 24 hours. - If medical issues evolved during patient’s ED stay where they were no longer safe to discharge, and no longer required physical therapy evaluation, then they were removed from observation and their disposition was adjusted accordingly. - The physical therapist gave recommendations regarding patient’s suitability for rehabilitation placement or home rehabilitation services. - Case management services available weekdays 7 am -10 pm (reduced hours on weekends and holidays) worked with patients and their families to identify the best services for them. - Case management facilitated applications to the patient’s insurance provide to reimburse for recommended rehabilitation placement. - Off-hours consults for physical therapist and case management services were noted on a shared electronic medical record space. | ED length of stay by disposition category  Rate of case management consultation by chief complaint  Disposition by chief complaint  72hr ED bounce-back resulting in admission by chief complaint. | - ED median length of stay based on disposition   - Admitted 24.3hrs   - Discharged home without services 16.8hrs   - Discharged home with services 19.1hr   - Discharged to rehabilitation 28.6 hr - Case management consulted mostly in patients with falls (95%), weakness (94%), and cardiopulmonary (94%). - Highest proportion of discharges home with services were for cardiopulmonary issues (42%), gastrointestinal illness (38%), and neurologic chief complaints (36%). - Patients with back pain had highest proportion of discharges home without services (27%) - Fall and general weakness had the highest proportion of discharge to rehabilitation (35% and 37%, respectively) - 4 patients discharged home without additional services re-presented to the ED within 72 hours and among them 3 were admitted. - Among those discharged home with services, 33 represented and 13 were admitted. - Of those discharged to rehabilitation, 6 represented, and 1 was admitted.   Limitations:   - Single-site study/ - Despite physiotherapist involvement, patient’s disposition was not always within the control of the physiotherapist. |
| 1. 5 | Guttman et al., 2004  Academy Emergency Medicine  Canada | *Intervention*: ED-based discharge planning program   - Three ED-based nurse discharge plan coordinators (NDPC) were selected and provided with a one-week intensive orientation conducted by a nurse specialist. - Training topics included geriatric assessment and health needs of community-dwelling elders, visits to community health centers, patient home visits, and problem-solving of common medical issues over the telephone. - NDPC assessed the patient’s perception of managing the health problem, collaborated in strategies for managing concern and treatment recommendations, use of written medication and symptom management tools and facilitated referral or liaison with community health care professionals as needed - NDPC provided telephone follow-up 24 hours post discharge, and was available to consult with patients and their family members up to one week following the emergency visit. - Weekly meetings with the study coordinator were organized during the course of the study | Rate of unscheduled return visits at 8 and 14days.  Hospitalization rate on revisit within 14 days  Satisfaction with discharge information provided  Rate of patient reported adherence to new medications  Rate of patient reported wellbeing at day 8 | - NDPC were less likely to have unscheduled revisits to the ED during their first 14 days of discharge when compared with usual care. However, was not statistically significant when adjusted for all covariates. - Reduction in unscheduled admissions within 14 days of ED discharge by 17% but no statistical difference due to low number in both control and intervention group. - Intervention group reported high levels of satisfaction in discharge process, but not specifically discharge information. - 90% of patients reported adherence to new medication - No rate of patient-reported wellbeing reported.   Limitations:   - Issues with balancing selection bias with study design causing particularly because there was no ability to blind patients or families from the interventions. - Some of the patient satisfaction tools used to collect data were not validated. - Likely selection bias towards sicker and frailer patients which affected comparison. - Single-site study. - Limited resources meant that target population was much narrower than originally intended. |
| 1. 6 | Hardy et al., 2001  Emergency Medicine Journal  UK | *Intervention*: A & E based approach   - A project team based in the A & E department analyzed baseline data to identify a group of patients for intervention. A comparison of this group was made with the historical control - Using eligibility criteria, an ED-based clinical assistant (3.5 hours daily) and nurse (7.5 hours daily) six days a week (Mon-Sat) identified suitable patients in the A & E department or among those admitted to the observation, department of medicine for the elderly or trauma wards. - While in-hospital patient’s medical needs were met, and fast-track occupational and physiotherapy assessments were arranged where appropriate. - Suitable patients were discharged home with support from a rapid response community team (RRCT). The RRCT consisted of a district nurse coordinator, supported by health care assistants (30hrs/week) who took referrals, organized required care and ’onitored patients' progress. Community therapists were also available when needed. - The team provided assistance with activities of daily living as well as rehabilitation, mobilization, and simple exercises for up to two weeks. - After 2 weeks, patients were either discharged from the RRCT program or transferred to social service support using established referral pathways. - District nurses visited project patients within 24 hours of discharge from the hospital. | Length of hospital stay  Hospital re-admission rates.  Patient reported satisfaction of hospital and community care, adequacy of analgesia, understanding of explanations. | - Liaison between the hospital and community teams was highly effective for early discharges and admission avoidances. - The average length of stay of the intervention group was 1.7 days and of control group was 6.3 days. - Readmission rate for intervention group was 1.2% and that for control group was 1.5% - 97% patients were satisfied with their discharge arrangements from hospital. - The cost savings for intervention group alone would be around £116 450.   Limitations:   - Patient satisfaction survey was limited and response rate was low. - Could not accurately assess number of bed days in hospital saved due to expansion of the program to include services not measured in the study. - Single-site study. |
| 1. 7 | Heeren et al., 2019  BMC Geriatrics  Belgium | *Intervention*: Unplanned Readmission prevention by Geriatric Emergency Network for Transitional care (URGENT) care model   - URGENT care model included four (4) consecutive steps to usual care and was led by a geriatric emergency nurse. - Older adults at risk for adverse events were identified using a validated screening tool and clinical judgement. High risk patients were included, low-risk patients were only included if ED staff estimated that patient would benefit. - Comprehensive geriatric assessment (CGA) was conducted for high-risk patients. - CGA-based multidisciplinary plan was tailored to meet the patient’s needs, capacity, and preferences - Geriatric follow-up provided if necessary. - A community nurse or social worker, supported by CGA-based advice, provided free community case manager follow-up for patients at risk for adverse events who were not hospitalization after index ED visit. - The geriatric emergency nurse was authorized to refer eligible patients to the facility-based geriatric day clinic for in-depth assessment and medical evaluation. | All outcomes measured at 30 and 90 days after discharge.  Unplanned ED readmission rate  ED length of stay  Hospitalization rate  In-hospital length of stay  Rates of higher level of care (i.e. professional organized living arrangement that differed from patient’s usual living place in the community)  Post-hospitalization mortality.  Functional decline | - Unplanned re-admission rate for control and intervention group:   - At 30 days: 12.1% and 13.1% respectively.   - At 90 days: 22.1% and 23.9% respectively - Median ED length of stay:   - Control group 19.1   - Intervention group 12.7      - 70% of intervention group was hospitalized compared with 67.7% control group which was statistically significant after comparison correction.   Median Hospital length of stay   - - Control group 8.7 days   - Intervention group 8.6 days - Higher level of care was comparable for both cohorts at follow-up (approx. 14% at hospital discharge and approx. 7% at both 30 and 90 days) - 6.4% in control group and 5.6% in intervention group died ninety days after hospital discharge. - Patient with death post-hospitalization were excluded. - No differences between the control and intervention cohort for functional decline.   Limitations:   - Risk-stratification tool still required some validation at the time of the study. - Selection bias was a concern, especially because including a diverse sample of patients was difficult due to consent process for those with cognitive disabilities. - The department moved during the study, it’s unknown if that move impacted results. - Single-site study. |
| 8. | Miller et al., 1996  Journal of the American Geriatrics Society  US | *Intervention*: Geriatric Case-Finding and Liaison (GCFL) service   - A nurse, trained in comprehensive geriatric assessment (CGA) and assessment instruments employed in the study, established referral and information transfer arrangements with a wide variety of community-based clinical and social services. - The nurse performed geriatric assessments (approximately 35min in length) to older patients visiting ED from noon-8p.m. on alternate dates. - Intervention-eligible patients were recruited and consented. A control patient of a similar gender and age (+/- 5 years) was identified for each intervention patient using the “matching day’s” log book. Control patients not contacted initially. - Each patient assessment included basic descriptive information, functional ability, severity of illness, cognition status, depressive symptoms, nutritional status, bowel and bladder habit, and any medical contraindications. - All the assessments and recommendations (further work-up, evaluations, referrals) were recorded using a ‘Geriatric Concerns and Suggestions’ sheet and attached to the ED chart for ED staff. A summary report was attached to the patient chart. Suggestions were discussed with patient and family. - Patient or caregiver telephone follow-up provided to those discharged from the ED at 7 to 10 days to assist with referrals and treatment plans, as needed, and to answer questions. - Eligible in-patients were provided follow-up by the CGA nurse assess if the identified issues were addressed, to advocate for follow-through, and to arrange follow-up for the patient if desired by the care team. - Both the intervention and control patients were called 3 months after their index ED visit to evaluate their health status and use of services in the interim. - Information obtained via proxy if the patient could not respond to assess impact of the intervention on outcome measures during the intervening 3 months since the index visit. | At 3 months:  Living situation  (Home, apartment or nursing home)  Rate of dependency in basic ADLs and iADLs.  Number of visits to physicians  Number of visits to EDs  Length of hospital stay  Quality of Life (0-100 scale)  Number of new dental or social services.  Number of new advance directive plans  Length of stay in ED (min) | - An average of 2.7 geriatric concerns per subject were evaluated in the ED. - Rates of living at home or in an apartment in discharged intervention groups (62%) versus discharged control group (70%).   - Difference in subgroup aged 75yrs and older between living at home (54%) at follow-up when compared to the control group (67%) that approached statistical significance. - No difference between the two groups in ability to climb stairs incontinence, levels of alertness, change in weight to follow-up. Intervention group demonstrated slightly higher prevalence of difficulty communicating. - Higher number of visits to a physician in the discharged intervention group (6.3) versus control group (4.5). - Trend towards fewer visits to EDs in the intervention group, not statistically significant. - Hospital length of stay in the discharged intervention group compared with discharged control group was 5.0 nights and 5.3 nights respectively. - ED staff complied with 61.6% of suggestions provided by the GCFL nurse about 275 of the patients - Minimal increase of statistical significance in the discharged group in the number of newly instituted dental/social services. - No difference in self-rated health or reported sadness or depression. - Trend towards more newly developed advance directives, but not statistically significant. - Intervention group spent more time in ED compared to the control group   Limitations:   - Several challenges as a result of health policy that made it difficult to coordinate care with competing providers. - Single-site study, only one nurse was available and |
| 9. | Morse et al., 2019  Geriatrics  US | *Intervention*: Geriatric Emergency Department Innovation (GEDI) program   - Trained Geriatric Nurse Liaisons (GNLs) identified older adults through the Identify Seniors at Risk (ISAR) tool and further assessed functional ability, cognitive status, caregiver strain, and transition readiness. - Patients were assessed by the GNLs and determined if they could receive optimal home-treatment with outpatient follow-up without being admitted to the hospital. - GNLs designed a discharge care plan before the patient’s discharge from the ED. Referrals to primary care and specialist services were done as needed. - After discharge, GNL made follow-up phone calls at two time points to assess needs and answer questions. | Description of patient concerns when followed-up at 24-72hrs and 10-14 days post ED discharge  Description of nurse responses when providing follow-up at 24-72hrs and 10-14 days post ED discharge | - The emergent themes in the patient concerns included clinical symptoms, medication questions, medical equipment, therapy or home health services, and follow-up with specialists or primary care providers. - The themes in the nurse responses included providing clinical information, medication counseling, care coordination relating to appointments, and communication with social workers to arrange other social services - Concerns and new information were more frequently elicited at the later time-point with regard to physician follow-up appointment scheduling. - Nurse coordination of durable medical equipment, physical therapy, and home health nursing coordination took place more frequently at the 24-72 h follow up relative to the 10-14 day follow-up - Calls at the second time-point allowed nurses to connect patients with services that were identified as potentially beneficial upon the initial physician follow-up.   Limitations:   - Unable to provide analysis of differences between follow-up results due to nature of the study design and data collection method. |
| 10. | Poncia et al., 2000  Journal of Accident and Emergency Medicine  UK | *Intervention*: Nurse-led next-day telephone follow-up   - ED-based community liaison nurse (CLN) reviewed notes and referrals of all patients aged 75 or over discharged from the department and contacted patients by telephone the next day. Those who attended over the weekend were contacted on the following Monday. - CLN used semi-structured questions to assess current level of disability, clinical deterioration since discharge, type of care received at home, compliance with medication and patient comprehension. - Using a study-developed 6-level scoring system, patient were stratify into six categories based on the perceived level of risk. - Scoring as follows:   1 & 2 🡪 no issues identified.  3🡪Issues identified but non-urgent PHC assessment required.  4 🡪Additional medical or social issues that required more urgent investigation by PHC.  5🡪 At risk patients who required immediate medical or social problems.  6 🡪At risk patients with issues that required urgent consultation with ED consultant for possible review.   - After telephone assessment appropriate interventions were made. | Understanding the type of advice given by community liaison nurse during telephone follow-up at 24-72hrs hours.  Understanding the type of health care professional that visits patient at home | - CLN contacted 46% patients on first attempt and remainder required up to four telephone calls to make contact - 58% patients were supported at home by friends or family, 26% were supported by agencies, 11% had home help, 9% had support from community nurse, 9% from social services, and 1.5% received meals on wheels - CLN gave advice on a broad range of issues including medication, follow up, occupational therapy, diet, and dressings - 69% understood health education given by CLN, 83% understood the follow up advice given - Early follow up was arranged for those who failed to understand CLN advice - Existing home support was insufficient for 8%, immediate intervention was needed in another 8%, and 15% were taking incorrect or inadequate medication - 559 referrals were made in total - 23% patients were visited at home by their GP, health visitor, district nurse, community pharmacist or other agencies, and an emergency response was requested in six cases - Services accessed included Specialist services (e.g., stoma nurses), and support form local or national support and self- help group   Limitations:   - Poor documentation and application of knowledge to connect patients with community follow-up by ED staff meant that care gaps were left unmet. - High workload environment placed higher risk on social support needs being under-recognized and under-estimated often leading to escalation later on. - Single-site.. |
